# Surveillance Systems to Track Progress Toward Polio Eradication — Worldwide, 2015–2016

**DOI:** 10.15585/mmwr.mm6613a3

**Published:** 2017-04-07

**Authors:** Edmond F. Maes, Ousmane M. Diop, Jaume Jorba, Smita Chavan, Rudolph H. Tangermann, Steven G. F. Wassilak

**Affiliations:** ^1^Global Immunization Division, CDC; ^2^Polio Eradication Department, World Health Organization, Geneva, Switzerland; ^3^Division of Viral Diseases, CDC.

Global measures to eradicate polio began in 1988; as of 2014, four of six World Health Organization (WHO) regions have been certified polio-free. Within the two endemic regions (African and Eastern Mediterranean), Nigeria, Afghanistan, and Pakistan have never interrupted transmission of wild poliovirus (WPV) (*1*). The primary means of detecting poliovirus transmission is surveillance for acute flaccid paralysis (AFP) among children aged <15 years, combined with collection and testing of stool specimens from persons with AFP for detection of WPV and vaccine-derived polioviruses (VDPVs) (viruses that differ genetically from vaccine viruses and can emerge in areas with low vaccination coverage and cause paralysis) in WHO-accredited laboratories within the Global Polio Laboratory Network (*2*,*3*). AFP surveillance is supplemented by environmental surveillance for polioviruses in sewage from selected locations (*4*). Genomic sequencing of the VP1-coding region of isolated polioviruses enables mapping transmission by time and place, assessment of potential gaps in surveillance, and identification of the emergence of VDPVs. This report presents poliovirus surveillance data from 2015 and 2016, with particular focus on 20 countries in the African Region and six in the Eastern Mediterranean Region that reported WPV or circulating VDPVs (cVDPVs) during 2011–2016, as well as the three countries most affected by the 2014–2015 Ebola virus disease (Ebola) outbreak (Guinea, Liberia, and Sierra Leone). During 2016, 12 (60%) of the 20 African Region countries and all six of the Eastern Mediterranean Region countries met both surveillance quality indicators (nonpolio AFP rates of ≥2 per 100,000 persons aged <15 years per year and ≥80% of AFP cases with adequate stool specimens [stool adequacy]) at the national level; however, provincial-level variation was seen. To complete and certify polio eradication, surveillance gaps must be identified and surveillance activities, including supervision, monitoring, and specimen collection and handling, further strengthened.

## Acute Flaccid Paralysis Surveillance

The quality of AFP surveillance is measured by two principal indicators. The first is the nonpolio AFP (NPAFP) rate (i.e., the number of NPAFP cases per 100,000 children aged <15 years per year of observation). An NPAFP rate ≥2 is considered sufficiently sensitive to detect WPV or VDPV cases if poliovirus is circulating. The second indicator is the collection of adequate stool specimens from ≥80% of AFP cases, indicating that surveillance can effectively identify WPV and VDPV among persons with AFP (*3*). Stool adequacy refers to collection of two stool specimens ≥24 hours apart, within 14 days of paralysis onset, and the arrival of these specimens in good condition* at a WHO-accredited laboratory.

Among 47 African Region countries, 32,250 AFP cases were reported in 2016 and 26,052 in 2015. Although no WPV type 1 (WPV1) cases were reported in the African Region in 2015, all four WPV1 cases that occurred in the African Region in 2016 were reported from Nigeria (*5*). Eighteen cVDPV cases were reported in the African Region during 2015, including eight cVDPV type 2 (cVDPV2) cases (one from Nigeria and seven from Guinea) and 10 cVDPV type 1 (cVDPV1) cases (all from Madagascar). During 2016, only one cVDPV case was reported in the African Region, a cVDPV2 case from Nigeria (Table 1). Among the 20 countries evaluated in the African Region, 12 met both of the national surveillance indicators in 2016 compared with 10 in 2015. Among the three countries most affected by Ebola (Guinea, Liberia, and Sierra Leone), only Guinea met the NPAFP indicator and only Liberia met the stool adequacy indicator in 2015; however, because of insufficient clinical knowledge about how to exclude Ebola virus from clinical specimens, nearly all stool specimens from 2015 were untested and destroyed. In 2016, all three of the Ebola-affected countries had NPAFP rates ≥2, but only Guinea also achieved ≥80% stool adequacy.

**TABLE 1:** National and subnational acute flaccid paralysis (AFP) surveillance indicators and number of confirmed wild poliovirus (WPV) and circulating vaccine-derived poliovirus (cVDPV) cases, by country, for all countries with poliovirus transmission during 2011–2016 or that were affected by the Ebola outbreak in West Africa within the World Health Organization (WHO) African Region and Eastern Mediterranean Region, 2015 and 2016*

WHO Region/Country	No. AFP cases (all ages)	Regional/National NPAFP rate^†^	Subnational areas with NPAFP rate ≥2^§^ (%)	Regional/National AFP cases with adequate specimens^¶^ (%)	Subnational areas with ≥80% adequate specimens (%)	Population in areas meeting both indicators** (%)	No. confirmed WPV cases*	No. confirmed cVDPV cases*^,††^
**2015**								
**AFR (all 47 countries)^§§^**	**26,052**	**6.2**	**NA**	**90**	**NA**	**NA**	**0**	**18**
**Countries reporting WPV or cVDPV transmission during 2011–2016 and Ebola-affected countries (Guinea, Liberia, and Sierra Leone)**								
Angola	414	3.8	100	95	100	100	0	0
Cameroon	619	5.6	100	83	80	67	0	0
CAR	81	3.9	71	80	43	34	0	0
Chad	433	6.6	100	87	78	87	0	0
Cote d'Ivoire	353	4.0	85	90	80	71	0	0
DRC^¶¶^	2,117	6.0	100	74	9	6	0	0
Equatorial Guinea	9	2.9	43	22	0	0	0	0
Ethiopia^¶¶^	1,179	2.8	82	76	45	29	0	0
Gabon^¶¶^	61	8.6	100	33	0	0	0	0
Guinea	146	2.7	75	75	38	26	0	7
Kenya	619	3.1	89	85	74	68	0	0
Liberia	22	1.2	60	95	60	44	0	0
Madagascar	522	4.8	95	59	9	17	0	10
Mali	247	3.2	78	84	67	79	0	0
Mozambique	321	2.4	90	80	60	49	0	0
Niger^¶¶^	214	2.1	63	61	0	0	0	0
Nigeria	13,970	17.1	100	98	100	100	0	1
Republic of the Congo^¶¶^	117	5.3	100	78	45	29	0	0
Sierra Leone	41	1.5	50	79	25	23	0	0
South Sudan	331	6.5	100	94	90	90	0	0
EMR (all 21 countries)***	**13,215**	**6.4**	**NA**	**90**	**NA**	**NA**	**74**	**2**
**Countries reporting WPV or cVDPV transmission during 2011–2016**								
Afghanistan	2,738	18.9	100	93	94	94	20	0
Iraq	520	3.7	84	82	58	49	0	0
Pakistan	5,814	9.3	100	87	75	97	54	2
Somalia	281	5.4	100	96	100	100	0	0
Syria^†††^	236	3.1	57	85	71	43	0	0
Yemen	537	5.4	96	91	87	95	0	0
**2016**								
**AFR (all 47 countries)^§§^**	**32,250**	**7.5**	**NA**	**90**	**NA**	**NA**	**4**	**1**
**Countries reporting WPV or cVDPV transmission during 2011–2016 and Ebola-affected countries (Guinea, Liberia, and Sierra Leone)**								
Angola	396	3.5	94	94	100	84	0	0
Cameroon	871	7.9	100	85	90	82	0	0
CAR^¶¶^	143	7.0	100	73	43	40	0	0
Chad	484	7.2	100	83	72	76	0	0
Cote d'Ivoire	371	4.2	85	93	85	74	0	0
DRC^¶¶^	1,827	5.1	100	79	46	53	0	0
Equatorial Guinea	3	1.0	14	33	0	0	0	0
Ethiopia^¶¶^	1,048	2.5	82	78	36	8	0	0
Gabon^¶¶^	43	6.1	100	28	10	3	0	0
Guinea	1,065	20.1	100	87	88	85	0	0
Kenya	553	2.7	87	89	77	68	0	0
Liberia	69	3.5	87	75	47	40	0	0
Madagascar	788	7.6	95	85	77	81	0	0
Mali	307	3.8	89	89	78	96	0	0
Mozambique	426	3.3	100	82	50	65	0	0
Niger^¶¶^	366	3.5	88	63	0	0	0	0
Nigeria	17,837	21.2	100	98	100	100	4	1
Republic of the Congo	82	3.7	82	82	73	78	0	0
Sierra Leone	68	2.6	100	76	50	45	0	0
South Sudan	323	6.3	90	91	80	70	0	0
EMR (all 21 countries)***	**15,956**	**7.7**	**NA**	**90**	**NA**	**NA**	**33**	**1**
**Countries reporting WPV or cVDPV transmission during 2011–2016**								
Afghanistan	2,903	20.0	100	92	97	99	13	0
Iraq	605	4.2	89	80	63	48	0	0
Pakistan	7,797	12.5	100	88	88	99	20	1
Somalia	316	5.9	100	99	100	100	0	0
Syria^†††^	303	3.9	71	79	43	28	0	0
Yemen	715	7.1	100	91	91	97	0	0

**Abbreviations:** AFR = African Region; CAR = Central African Republic; DRC = Democratic Republic of the Congo; Ebola = Ebola virus disease; EMR = Eastern Mediterranean Region; NA = not applicable; NPAFP = nonpolio AFP.

* Data as of February 12, 2017.

^†^ Per 100,000 persons aged <15 years per year.

^§^ For all subnational areas regardless of population size.

^¶^ Standard WHO target is adequate stool specimen collection from ≥80% of AFP cases, assessed by timeliness and condition. In this analysis, timeliness was defined as two specimens collected ≥24 hours apart (≥1 calendar day in this data set), and both within 14 days of paralysis onset. Condition was defined as specimens arriving in good condition (reverse cold chain maintained and received without leakage or desiccation) in a WHO-accredited laboratory.

** Percent of the country’s population living in subnational areas which met both surveillance indicators (NPAFP rates of ≥2 per 100,000 persons aged <15 years per year and ≥80% of AFP cases with adequate specimens).

^††^ cVDPV was associated with two or more cases of AFP with genetically linked VDPVs. Guidelines for classification of cVDPV changed in 2015 and can be found at http://polioeradication.org/wp-content/uploads/2016/07/VDPV_ReportingClassification.pdf.

^§§^ Algeria, Angola, Benin, Botswana, Burkina Faso, Burundi, Cameroon, Cabo Verde, Central African Republic, Chad, Comoros, Congo, Cote d’Ivoire, Democratic Republic of Congo, Equatorial Guinea, Ethiopia, Eritrea, Gabon, Gambia, Ghana, Guinea, Guinea-Bissau, Kenya, Lesotho, Liberia, Madagascar, Malawi, Mali, Mauritania, Mauritius, Mozambique, Namibia, Niger, Nigeria, Rwanda, Sao Tome and Principe, Senegal, Seychelles, Sierra Leone, South Africa, South Sudan, Swaziland, Togo, Uganda, Tanzania, Zambia, and Zimbabwe.

^¶¶^ Stool adequacy dropped to <80% when stool condition was included with timeliness. Timeliness was defined as two specimens collected ≥24 hours apart (≥1 calendar day in this data set), and both within 14 days of paralysis onset. Condition was defined as specimens arriving in good condition (reverse cold chain maintained and received without leakage or desiccation) in a WHO-accredited laboratory.

*** Afghanistan, Bahrain, Djibouti, Egypt, Iran, Iraq, Jordan, Kuwait, Lebanon, Libya, Morocco, Oman, Pakistan, Qatar, Saudi Arabia, Somalia, Sudan, Syria, Tunisia, United Arab Emirates, and Yemen.

^†††^ The NPAFP rate for Syria is artificially low because of displaced populations and the lack of official data from areas not under government control.

Among 21 Eastern Mediterranean Region countries, 13,215 AFP cases were reported in 2015, and 15,956 in 2016. Two Eastern Mediterranean Region countries (Afghanistan and Pakistan) reported WPV1 cases in 2015 (n = 74) and 2016 (33). The number of WPV1 cases reported by Afghanistan declined from 20 in 2015 to 13 in 2016; the number reported from Pakistan declined from 54 (2015) to 20 (2016). Two cVDPV2 cases were reported from the region in 2015 compared with one in 2016; all three cVDPV2 cases were reported from Pakistan. All six Eastern Mediterranean Region countries reviewed met both surveillance indicators in 2015 and 2016; however, national-level surveillance indicators masked subthreshold surveillance performance at subnational levels in both regions (Table 1) (Figure).

**FIGURE:**
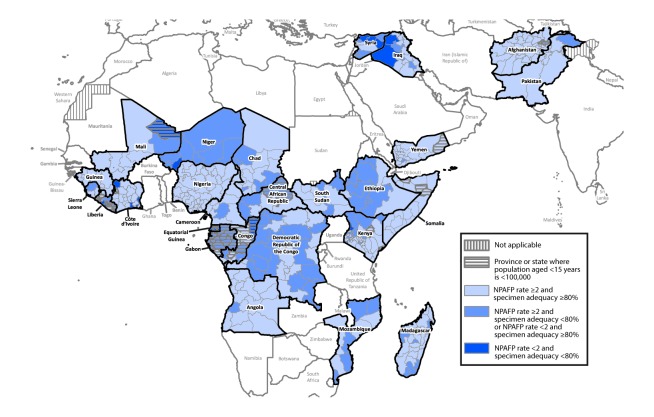
Combined performance indicators for the quality of acute flaccid paralysis surveillance* in subnational areas (states and provinces) of 26 countries that had poliovirus transmission during 2011–2016 or were affected by the Ebola outbreak in West Africa during 2014–2015 — World Health Organization African and Eastern Mediterranean Regions, 2016^†^ **Abbreviations:** AFP = acute flaccid paralysis; NPAFP = nonpolio AFP. * The Global Polio Eradication Initiative has set the following targets for countries with current or recent wild poliovirus transmission and their states/provinces: 1) NPAFP detection rate of ≥2 cases per 100,000 persons aged <15 years per year, and 2) adequate stool specimen collection from ≥80% of AFP cases, with specimen adequacy assessed by timeliness and condition. Timeliness was defined as two specimens collected ≥24 hours apart (≥1 calendar day) and both within 14 days of paralysis onset. Good condition was defined as specimens arriving without leakage or desiccation in a maintained reverse cold chain at a World Health Organization–accredited laboratory. ^†^ Data are for AFP cases with onset during 2016, reported as of February 14, 2017.

## Environmental Surveillance

Testing of sewage samples supplements AFP surveillance by identifying poliovirus transmission that might occur in the absence of detected AFP cases (*4*). In April 2016, all OPV-using countries switched from using trivalent OPV (tOPV) to bivalent OPV (bOPV), containing vaccine virus types 1 and 3, to reduce circulation of type 2 vaccine virus, which is responsible for most cVDPVs (*6*). Testing sewage is useful for monitoring the decline of oral poliovirus vaccine (OPV) type 2-related poliovirus (OPV2) in the environment after the global switch. The number of environmental surveillance collection sites increased within Afghanistan, Nigeria, and Pakistan from 21 at the end of 2011 to 138 as of February 2017. Frequency of sample collection also affects the ability to detect virus. Environmental surveillance is conducted in 34 countries without recent active WPV transmission, including nine on the African continent.

In Nigeria, sewage sampling is conducted at 57 sites in 15 states and the Federal Capital Territory. No WPVs have been isolated from sewage since May 2014, when WPV1 was isolated from one sample in Kaduna State. Low-level transmission of a cVDPV2 that emerged in Nigeria in 2005 and of a cVDPV2 that originated in Chad in 2012 was documented from samples collected during 2015–2016; the most recent cVDPV2 was detected from specimens collected in Borno State in March, 2016. Environmental sampling in Afghanistan is conducted at 15 sites in five provinces at high risk for WPV transmission. WPV1 was detected in samples collected in all five provinces in 2015 and in two provinces (Hilmand and Nangarhar) in 2016. In Pakistan, sampling is conducted at 62 sites in five provinces/regions, including 25 new sites in 2016. The proportion of samples testing positive for WPV1 significantly decreased (p<0.001) from 19.6% (86/439) in 2015 to 10.6% (69/648) in 2016. WPV1 was detected in all five provinces/regions in both years.

## Global Polio Laboratory Network

The Global Polio Laboratory Network consists of 146 WHO-accredited poliovirus laboratories in all WHO regions. Global Polio Laboratory Network member laboratories follow standardized protocols to 1) isolate and identify poliovirus, 2) conduct intratypic differentiation to identify WPV or screen for Sabin (vaccine) poliovirus and VDPVs (*7*), and 3) conduct genomic sequencing. Sequencing results help monitor pathways of poliovirus transmission by comparing the nucleotide sequence of the VP1-coding region of poliovirus isolates. To meet standard laboratory timeliness indicators for stool specimen processing, laboratories should report ≥80% of poliovirus isolation results within 14 days of specimen receipt, ≥80% of intratypic differentiation results within 7 days of isolate receipt, and ≥80% of sequencing results within 7 days of intratypic differentiation results. The standard programmatic indicator combining field and laboratory performance is to report intratypic differentiation results for ≥80% of isolates from AFP cases within 60 days of paralysis onset. This indicator considers the entire interval from paralysis onset to specimen testing. The accuracy and quality of testing at Global Polio Laboratory Network member laboratories is monitored through an annual accreditation program that includes onsite reviews and proficiency testing.

Global Polio Laboratory Network laboratories met timeliness indicators for poliovirus isolation for both years in all regions except the European Region in 2015 (Table 2). The overall timeliness indicator for onset to intratypic differentiation results was met in both years in all regions except the European Region in 2015. The Global Polio Laboratory Network tested 192,250 stool specimens in 2015 and 220,920 in 2016. WPV1 was isolated from 74 AFP case specimens in 2015 and from 37 AFP case specimens in 2016. In addition, cVDPV was detected in 33 AFP case specimens in 2015 and 11 AFP case specimens in 2016.

**TABLE 2:** Number of poliovirus isolates from stool specimens of persons with acute flaccid paralysis and timing of results, by World Health Organization (WHO) region, 2015 and 2016*

WHO Region/Year	No. specimens	No. poliovirus isolates			% Poliovirus isolation results on time^¶^	% ITD results within 7 days of receipt at laboratory**	% ITD results within 60 days of paralysis onset
		Wild	Sabin^†^	cVDPV^§^			
**African**							
2015	50,960	0	3,579	18	82	79	95
2016	65,520	4	4,771	4	95	94	97
**Americas**							
2015	1,698	0	44	0	84	100	100
2016	4,246	0	18	0	84	92	91
**Eastern Mediterranean**							
2015	25,827	74	951	2	93	99	95
2016	31,928	33	1,612	1	94	98	98
**European**							
2015	3,655	0	106	4	63	93	70
2016	3,480	0	71	0	82	100	86
**South-East Asia**							
2015	96,783	0	3,335	2	97	86	98
2016	101,550	0	5,247	2	98	99.5	99
**Western Pacific**							
2015	13,327	0	194	7	96	98	86
2016	14,196	0	253	4	96	98	96
**Total^††^**							
**2015**	**192,250**	**74**	**8,209**	**33**	**89**	**85**	**96**
**2016**	**220,920**	**37**	**11,972**	**11**	**96**	**97**	**98**

**Abbreviations**: cVDPV = circulating vaccine-derived poliovirus; ITD = intratypic differentiation.

* Data as of February 14, 2017.

^†^ Either 1) concordant Sabin-like results in ITD test and VDPV screening, or 2) ≤1% VP1 nucleotide sequence difference compared with Sabin vaccine virus (≤0.6% for type 2).

^§^ For poliovirus types 1 and 3, 10 or more VP1 nucleotide differences from the respective poliovirus; for poliovirus type 2, six or more VP1 nucleotide differences from Sabin type 2 poliovirus.

^¶^ Results reported within 14 days for laboratories in the following WHO regions: African, Americas, Eastern Mediterranean, and South-East Asia, and Western Pacific. Results reported within 28 days for the European Region.

** Results of ITD reported within 7 days of receipt of specimen.

^††^ For the last three indicators, total represents weighted mean percent of regional performance.

In 2016, the West Africa B1 (WEAF-B1) genotype was isolated in Nigeria, where it had last been detected in 2014. In Afghanistan and Pakistan, the only genotype isolated in 2016 was South Asia (SOAS); this was the only genotype isolated worldwide in 2015. Overall genetic diversity declined among WPV1 isolates in 2016.

A poliovirus isolate with ≥1.5% nucleotide divergence in genomic sequencing of the VP1-coding region compared with previous isolates is called an “orphan” virus; orphan viruses indicate prolonged undetected virus circulation and gaps in AFP surveillance. In 2016, as in 2015, genomic sequencing indicated that WPV1 and cVDPV cases were likely missed by AFP surveillance. Orphan WPV1 isolates were associated with one of 20 WPV1 cases reported from Pakistan and three of four WPV1 cases reported in Nigeria in 2016. Orphan cVDPVs were isolated from stool specimens of AFP patients in four countries (Pakistan, Afghanistan, Nigeria, and Cameroon) in 2015; in 2016, only Nigeria reported an orphan cVDPV virus from a stool specimen of an AFP case contact in Borno State.

Three countries outside the African and Eastern Mediterranean Regions reported cVDPVs in 2015: Ukraine (cVDPV1), Laos (cVDPV1), and Myanmar (cVDPV2). No additional VDPV cases were detected in Ukraine or Myanmar in 2016; the last case in Laos had onset in January 2016.

## Discussion

The number of reported WPV cases declined to the lowest point ever in 2016. Although the majority of national-level surveillance quality indicators improved in 2016, considerable variation was seen at subnational levels. Despite meeting surveillance indicator standards for several years at the state level in Nigeria, the discovery of previously undetected circulation of individual WPV lineages for several years as well as continued inaccessibility of certain geographical areas with underimmunized persons has raised concerns (*5*), prompting detailed reviews of surveillance and geographic accessibility. Although conflict has limited access in several areas (including Somalia, South Sudan, and Syria), effective community-based surveillance provides some assurance of the absence of poliovirus circulation in many of those areas.

Certification of polio-free status requires at least 3 years of timely and sensitive polio surveillance (*8*), including timely stool collection, and timely and appropriate transport of specimens to the laboratory. Specimen condition was a particular concern in the Democratic Republic of the Congo, Ethiopia, Gabon, Madagascar, and Niger in 2016. With the end of the Ebola outbreak, polio surveillance performance is improving in West Africa, although more work remains to return to pre-outbreak surveillance quality indicators.

The findings in this report are subject to at least two limitations. First, the surveillance indicators do not fully reflect security-related issues, issues associated with mobile and difficult-to-access populations, or other factors that affect surveillance performance. For example, in Iraq and the Syria, population movements related to conflict make interpretation of AFP surveillance indicators difficult. Second, high NPAFP rates do not necessarily imply sensitive surveillance, because a proportion of reported AFP cases might not be actual AFP cases, and not all actual AFP cases might be detected.

Supervision and monitoring of AFP surveillance can help ensure that all actual AFP cases are identified, reported, and appropriately investigated. As polio case counts decrease, maintenance of sensitive AFP surveillance becomes increasingly critical. Environmental surveillance has been an important supplement to AFP surveillance, and when carefully conducted, can improve detection of circulating virus, particularly in areas at high risk with suboptimal AFP surveillance. The risk for WPV and cVDPV importation and for cVDPV emergence exists even in countries in polio-free regions. To achieve polio eradication, surveillance performance should be closely monitored and quality should be maintained globally to promptly identify and respond to all cases of polio.

**Summary**

**What is already known about this topic?**

Surveillance is a cornerstone of polio eradication programs. Acute flaccid paralysis (AFP) surveillance is the primary means of poliovirus detection, supplemented by environmental surveillance (i.e., the collection of sewage samples for poliovirus testing) to identify poliovirus circulation in the absence of detected AFP cases.

**What is added by this report?**

Although surveillance performance indicators are improving, gaps remain, including substantial variation at subnational levels (i.e., in 2016, of 20 African Region countries, 19 met the NPAFP target at the national level versus 11 at all subnational levels). The number of environmental surveillance locations has increased substantially (from 21 at the end of 2011 in Afghanistan, Nigeria, and Pakistan to 138 as of February 2017) and has enhanced the ability to detect poliovirus circulation and possible AFP surveillance gaps. In countries previously affected by Ebola, surveillance quality is improving, although further measures are needed to reach preoutbreak levels.

**What are the implications for public health practice?**

Rapid improvements in AFP surveillance are needed in several African Region countries to ensure timely certification of polio-free status.  Gaps in surveillance quality, especially at the subnational level, need to be identified and resolved through well-supervised active and monitored passive surveillance, and supplemental environmental and virologic surveillance. As long as polioviruses continue to circulate in any country, all countries remain at risk.

## Footnotes

* Reverse cold chain maintained and received without leakage or desiccation at a WHO-accredited laboratory. Reverse cold chain is maintained when stool specimens are stored at 4°–8°C (32°–39°F) immediately after collection, frozen at −20°C (−4°F) when received for processing, and shipped to a WHO-accredited laboratory in dry ice or cold packs. Freezing of specimens is unnecessary if specimens can be received at a WHO-accredited laboratory within 72 hours of collection.
